# Knowledge and Perceptions of Couples' Voluntary Counseling and Testing in Urban Rwanda and Zambia: A Cross-Sectional Household Survey

**DOI:** 10.1371/journal.pone.0019573

**Published:** 2011-05-09

**Authors:** April L. Kelley, Etienne Karita, Patrick S. Sullivan, Francois Katangulia, Elwyn Chomba, Michel Carael, Joseph Telfair, Steve M. Dunham, Cheswa M. Vwalika, Michele G. Kautzman, Kristin M. Wall, Susan A. Allen

**Affiliations:** 1 Project San Francisco, Rwanda Zambia HIV Research Group, Kigali, Rwanda; 2 Department of Epidemiology, Rollins School of Public Health, Emory University, Atlanta, Georgia, United States of America; 3 Social Scientific Systems, Inc/Monitoring and Evaluation Management Services (SSS/MEMS), Kigali, Rwanda; 4 Zambia Emory HIV Research Project, Rwanda Zambia HIV Research Group, Lusaka, Zambia; 5 UNAIDS, Geneva, Switzerland; 6 Department of Public Health Research and Practice, Center for Social, Community and Health Research and Evaluation, University of North Carolina at Greensboro, Greensboro, North Carolina, United States of America; 7 Department of Pathology and Laboratory Sciences, School of Medicine, Departments of Epidemiology and Global Health, Rollins School of Public Health, Emory University, Atlanta, Georgia, United States of America; University of Cape Town, South Africa

## Abstract

**Background:**

Most incident HIV infections in sub-Saharan Africa occur between cohabiting, discordant, heterosexual couples. Though couples' voluntary HIV counseling and testing (CVCT) is an effective, well-studied intervention in Africa, <1% of couples have been jointly tested.

**Methods:**

We conducted cross-sectional household surveys in Kigali, Rwanda (n = 600) and Lusaka, Zambia (n = 603) to ascertain knowledge, perceptions, and barriers to use of CVCT.

**Results:**

Compared to Lusaka, Kigali respondents were significantly more aware of HIV testing sites (79% *vs.* 56%); had greater knowledge of HIV serodiscordance between couples (83% *vs.* 43%); believed CVCT is good (96% *vs.* 72%); and were willing to test jointly (91% *vs.* 47%). Stigma, fear of partner reaction, and distance/cost/logistics were CVCT barriers.

**Conclusions:**

Though most respondents had positive attitudes toward CVCT, the majority were unaware that serodiscordance between cohabiting couples is possible. Future messages should target gaps in knowledge about serodiscordance, provide logistical information about CVCT services, and aim to reduce stigma and fear.

## Introduction

Sub-Saharan Africa remains the region of the world most heavily impacted by the HIV epidemic, accounting for 67% of all people living with HIV and 75% of AIDS deaths in 2007 [1]. The majority of prevalent and incident infections in Africa are among cohabiting heterosexual couples [2], [3], [4]. Because partners within a couple may have different HIV statuses, monogamous relationships do not protect partners from HIV [5], [6], [7]. It is estimated that half to three-quarters of new HIV infections could be prevented by voluntary HIV counseling and testing services targeted toward cohabiting couples [8]. Despite this, health facilities offering HIV testing services in sub-Saharan Africa rarely accommodate couples, and few couples seek testing together [9].

Barriers to couples' voluntary HIV counseling and testing (CVCT) include stigma, discrimination, gender inequality, concerns about confidentiality, lack of knowledge about availability of CVCT services, and misconceptions about HIV serodiscordance (i.e., partners may assume that the other partner shares their serostatus) [6], [10], [11], [12], [13], [14], [15]. Lack of awareness of partners' serostatus may result in transmission within discordant couples because protective behaviors are not adopted [16], [17], [18].

Individuals who have undergone HIV voluntary counseling and testing (VCT) may fail to disclose their HIV status to their sexual partners [19]. In Africa, published disclosure rates to a sexual partner among HIV-infected individuals range from 16–79%, with lower rates of disclosure by women, largely due to fear of partner reaction [20], [21], [22], [23], [24], [25], [26]. CVCT, where both partners participate, share their results, and formulate risk-reduction plans with a trained professional, addresses the difficulties of disclosing such sensitive information [27] and has been shown to reduce rates of HIV transmission, sexually transmitted infections and unintended pregnancies [28], [29], [30], [31], [32]. CVCT, when incorporated into antenatal health programs, also leverages the prevention impact of mother-to-child transmission programs (PMTCT) [33], [34], [35]. Despite years of evidence for the beneficial impact and feasibility of CVCT [36], [37] and development of a CDC-sponsored curriculum for training CVCT providers [38], CVCT services have not been routinely integrated into existing HIV and family planning services in Africa. In preparation for a neighborhood randomized control trial of an intervention to increase uptake of CVCT services in Kigali, Rwanda and Lusaka, Zambia, we conducted a cross-sectional household survey to establish knowledge, perceptions, and barriers regarding CVCT among adults residing in the study neighborhoods.

## Methods

The Rwanda Zambia HIV Research Group (RZHRG) consists of Project San Francisco in Kigali and the Zambia Emory HIV Research Project in Lusaka. RZHRG conducted this cross-sectional household survey to establish knowledge, perceptions, and barriers to CVCT in collaboration with the Rwandan Ministry of Health and Social Scientific Systems Inc/Monitoring & Evaluation Management Services in Kigali, and with the Institute of Economic and Social Research of the University of Zambia in Lusaka. The survey took place in Kigali and Lusaka from February to April, 2004. Potential respondents were informed of the purpose of the study, their rights, and the voluntary nature of their participation. Fieldwork interviewers obtained written signed consent. This study was reviewed and approved by the Office for Human Research Protections and the Institutional Review Boards in Rwanda, Zambia, and Emory University, USA.

Nine neighborhoods in Kigali and eight in Lusaka were assessed as potential sites for the household survey. Three non-overlapping neighborhoods were selected within both Kigali's and Lusaka's administrative districts that were geographically separated and did not contain clinics with overlapping catchment areas. These neighborhoods were selected based on their comparable population sizes and infrastructure characteristics. Selected neighborhoods were randomized in anticipation of a neighborhood randomized controlled trial of an intervention to promote CVCT services. Two of the three neighborhoods were randomly selected to receive an active CVCT promotion intervention, and each contained one stand-alone CVCT site per neighborhood which was unaffiliated with government clinics. One neighborhood was randomly selected to serve as the control neighborhood. The interventions in the randomized controlled trial were administered after the household survey presented in this paper, and consisted of promotion of CVCT via influential network leaders (INLs) and influential network agents (INAs) with or without access to a mobile CVCT unit. The control neighborhoods did not have a fixed research site for CVCT, only contained government clinics offering regular services, and no CVCT promotions were conducted.

Households in each neighborhood were selected using a probability sampling approach, and participants within households were selected purposively as described below. Thus, we view our sample as a convenience sample, although neighborhoods and households were selected randomly. Within each selected neighborhood, researchers approached a community leader to inform him/her about the study and to seek permission to undertake the study in their area. Households were then systematically sampled using a random starting point and sampling interval of n = 3. Every 3^rd^ house moving in one cardinal direction was therefore selected for participation. If no one was home at a selected house, the next house was sampled as a replacement. Men aged 15–60 years and women aged 15–49 years were eligible to participate. The eligible age ranges for men and women were chosen to include adults at highest risk of HIV transmission and differ given that peak HIV prevalence rates for women occur at younger ages than for men. The first eligible adult contacted was invited to participate, without regard to whether they were the head of the household. Houses were preselected to request a male or female to ensure a 1:1 sex ratio, and surveys were alternatively administered between the two sexes. In the event that the sampled household did not have an eligible responder of the preselected sex, no one was selected from that house. No incentive was offered for participation.

The selection of either male or female respondents was initially alternated to obtain an equal number of men and women in each neighborhood. As the study progressed, staff evaluated the numbers of men and woman recruited and adjusted the male to female recruitment ratio to result in equal numbers of male and female respondents within each neighborhood. The target sample size of the survey in each city was 600 households (200 respondents per study neighborhood). The survey instrument consisted of 20 closed questions assessing demographics, knowledge of HIV testing and serodiscordance, attitudes about CVCT, and facilitators and obstacles to CVCT. Questions were administered orally via face-to-face interview in Kinyarwanda in Kigali and Nyanja or Bemba in Lusaka. Interviewer survey teams consisted of at least one coordinator and three interviewers. The coordinator performed quality control of data collection and sampling methods. Interviewers attended a two-day training on the questionnaire and the sampling methods and a two-day training on data entry and management using SPSS (version 15.0; Statistical Packages for the Social Sciences, Chicago, USA).

Comparisons were made between responses obtained in Kigali *vs.* Lusaka and between responses obtained between control *vs.* intervention neighborhoods within each city. Responses from Kigali and Lusaka were also analyzed by cohabitation status, education, and gender. Chi-square and Fisher's Exact tests were performed, as appropriate, to test for statistical significance of compared proportions. T-tests were used to compare mean ages. Because the objective of the study was to describe baseline population characteristics in preparation for a randomized controlled trial, and since there were no statistically significant differences between the intervention neighborhoods, we were not concerned with the potential effect of clustering within neighborhoods on evaluation of the study objective. Additionally, since we did not analyze the data as a probability sample, but instead a convenience sample, we therefore did not adjust for clustering of observations within communities. Data were analyzed with the SAS statistical package (version 9.1.3; Statistical Analysis Software, North Carolina, USA).

## Results

Table S1 presents a comparison of Kigali and Lusaka respondents in intervention and control neighborhoods within each city. The survey sample consisted of 1,203 respondents in Kigali (n = 600) and Lusaka (n = 603) with equal proportions of men and women in each city. Unless specified, all comparisons below are statistically significant, with two-tailed p-values presented in Tables S1, S2, S3. The mean age of respondents was 29 years. Lusaka respondents were almost twice as likely as Kigali respondents to be highly educated (56% *vs.* 31% with secondary education or higher). A similar proportion of respondents in Kigali (61%) and Lusaka (57%) were cohabiting. Compared to Lusaka, respondents from Kigali were more likely to know of a place to test for HIV (84% *vs.* 69%), to cite hospitals or health centers (65% *vs.* 36%) rather than stand alone VCT centers (17% *vs.* 33%) as places to test, to know the name of a nearby facility that offered VCT (79% *vs.* 56%), and to report having heard or knowing about HIV testing for couples (94% *vs.* 67%). The radio (64% *vs.* 31%), local health clinic (22% *vs.* 14%), family (13% *vs.* 7%), and church (13% *vs.* 9%) were more commonly reported as sources of information about CVCT in Kigali, while in Lusaka the television (26% *vs.* 8%) and friends (35% *vs.* 13%) were more commonly reported.

Almost twice as many respondents in Kigali compared to Lusaka reported knowing about the possibility of HIV serodiscordance (83% *vs.* 43%). Kigali respondents were also more likely to say that a married/cohabiting person testing alone should disclose their HIV results to their partner (90% *vs.* 77%), and that couples' testing is good (96% *vs.* 72%). More Kigali respondents compared to Lusaka respondents were willing to test with their spouse (91% *vs.* 47%); respondents in Lusaka were more likely to prefer testing alone (6% *vs.* 4%) or not testing at all (9% *vs.* 1%) compared to Kigali respondents. Lusaka respondents were more likely to report that couples' testing is bad (18% *vs.* 5%) or to have no opinion on couples' testing (10% *vs.* 0%) and were more likely to report that CVCT might break up the family (9% *vs.* 4% of all respondents) or lead to depression (6% *vs.* <1%) relative to Kigali respondents.

Both psychosocial and logistical obstacles were reported as barriers preventing couples from being tested together for HIV. Lusaka respondents reported stigma as the major obstacle to CVCT (51% *vs.* 29% in Kigali), while in Kigali, partner reaction was the most commonly cited reason (41% *vs.* 24% in Lusaka). Fear of partner reaction could include fear of reaction to the suggestion of testing and what that suggestion might imply, as well as fear of partner reaction to a positive test result. Kigali respondents were also more likely than those in Lusaka to report distance to the testing facility or cost of the test as a barrier to testing (23% *vs.* 10%). The most common reasons given for testing as a couple was to know one's test result, which was more frequently reported in Lusaka than in to Kigali (91% *vs.* 47%) and to plan for the future (35% in Lusaka *vs*. 33% in Kigali). The finding that 91% of respondents in Lusaka reported knowledge of one's *individual* test result as a reason for testing as a couple was notable, supporting the conclusion that most respondents did not recognize that CVCT offered prevention impact beyond that of individual VCT. Kigali respondents were more likely than Lusaka respondents to cite prevention of transmission between partners (25% *vs.* 14%) and prevention of mother-to-child transmission (25% *vs.* 14%) as reasons for CVCT.

In Kigali, respondents from intervention and control neighborhoods had similar age and educational attainment. Respondents in intervention neighborhoods were less likely to be cohabiting (59% *vs.* 67%), more likely to have heard about CVCT at their local health center (25% *vs.* 15%), less likely to have heard about CVCT at a church (11% *vs.* 19%), and more likely to report partner reaction as an obstacle to couples' testing (44% *vs.* 34%). Respondents in the intervention group were less likely to report treatment possibilities (27% *vs.* 37%) and knowledge of one's HIV test result (41% *vs.* 60%) as reasons for testing, but were more likely to report planning for the family's future as a reason to seek CVCT services (38% *vs.* 24%) relative to the control group. The proportion reporting ‘to prevent HIV transmission between partners’ was not different in Kigali control and intervention communities (23% *vs.* 27%).

In Lusaka, respondents in the control neighborhood were older (31 years *vs.* 28 years), more highly educated (66% *vs.* 51% with secondary education or higher), and more likely to be cohabiting with a partner (67% *vs.* 51%) compared to the intervention neighborhoods. Control neighborhood respondents were more likely than intervention neighborhood respondents to know of a place to test for HIV (79% *vs.* 64%), to cite stand-alone VCT centers as a place to test (53% *vs.* 23%), to know the name of a nearby testing location (76% *vs.* 46%), to cite radio, television, or the family as sources of information about CVCT, and to know about the possibility of discordant results between couples (49% *vs.* 40%). Control respondents were less likely to report hospital or health centers as places to test for HIV relative to intervention respondents (26% *vs*. 41%). The neighborhoods were similar in opinions about CVCT and willingness to test with a partner. Respondents residing in the Lusaka control neighborhood more often cited stigma as the major reason preventing couples from getting tested for HIV together (62% *vs.* 45%) while those in the intervention neighborhoods cited logistical obstacles of distance/cost (14% *vs.* 3%) or duration of the test/taking blood (10% *vs.* 5%) as barriers. Lastly, control neighborhood respondents were more likely to report prevention of HIV transmission between spouses as a reason for seeking couples' testing (19% *vs.* 12%).

Table S2 compares Kigali respondents stratified by cohabitation, education, and gender. Cohabiting respondents were more likely to know of a place to test for HIV, to indicate that HIV tests may be obtained at a hospital or health center, to know the name of a place nearby to test, to have heard about CVCT from a local health center, to respond that couples' joint HIV testing is good, and to cite partner's reaction as a barrier to CVCT compared to non-cohabiting respondents. Cohabiting couples were less likely to have heard about CVCT from a friend or family member, to believe couples' testing is not good because it may break up the family, and to cite stigma as a barrier to seeking CVCT services. Those with a secondary education or higher were more likely to know of a place to test for HIV, to report that testing may be obtained at a blood bank/family planning center/other, to know the name of a nearby place to test for HIV, and to have heard about CVCT from radio, television, or newspaper. More educated Kigali respondents were also more likely to know about the possibility of HIV serodiscordance among married or cohabiting couples, to believe that joint testing is good, and to cite partner reaction as a barrier to seeking CVCT services. More educated respondents were less likely to report distance to a heath facility or testing costs as barriers to CVCT. Women were more likely than men to cite hospitals or health centers as places to test for HIV, to cite local health clinics as sources of information regarding CVCT, and to report partner reaction as a barrier to CVCT. Men were more likely than women to report stand alone VCT center as places to receive testing, to cite radio as a source of information about CVCT, and to report stigma as a barrier to CVCT.

Table S3 compares Lusaka respondents by cohabitation, education, and gender. Cohabiting respondents were more likely than non-cohabiting respondents to report the local health clinic as a source of information about couples' testing, to report willingness to test with a spouse or partner, and to cite planning for the family's future as a reason to seek CVCT services. Cohabiting respondents were less likely to report stand-alone VCT centers as places for receiving HIV testing. Respondents with higher education were more likely than those with lower education to know of a place to test for HIV, to report stand alone VCT centers for HIV testing, to know the name of a nearby place to test, to know about CVCT, to have heard about CVCT via television, to know about the possibility of HIV serodiscordance, to believe that couples' testing is good, and to be willing to test with their partner. More educated respondents were less likely to report ‘it is not important/it is God's will' as a reason couples' testing is not good. Women in Lusaka were more likely than men to cite hospitals/local health centers as places for HIV testing, to cite local health clinics as a source of information about CVCT, to know couples could be discordant, to believe that a person testing alone should share their HIV results with their partner, and to report stigma as a barrier to CVCT. Men were more likely than women to report newspapers as a source of information about CVCT, to report distance to a health facility or cost of testing as a barrier to CVCT, and to cite preventing HIV transmission between partners as a reason couples may seek testing.

Knowledge and perceptions toward CVCT were also analyzed stratified by age. Older study participants were significantly (p<0.05) more likely to report knowing of a place to test for HIV, knowing the name(s) of places nearby to test, knowing about CVCT, and having heard about CVCT from the newspaper. Younger study participants were significantly (p<0.05) more likely to report knowing that serodiscordance is possible between couples, having heard about CVCT from a friend, citing stigma and fear of partner reaction as reasons preventing couples from getting tested together, and citing prevention of vertical transmission and planning for the family's future as reason to seek CVCT.

## Discussion

This community-based survey confirms that attitudes towards CVCT in two African capitals are generally favorable. However, many respondents did not know where to access CVCT services, and knowledge about serodiscordance was low, particularly in Lusaka, where only two-fifths of respondents knew serodiscordance was possible between couples. Strikingly, less than one quarter of respondents cited prevention of transmission between partners as a reason for CVCT. These gaps in knowledge highlight the need for increased interventions, promotional messages providing information on HIV serodiscordance and where to access CVCT services, and the importance of CVCT to prevent HIV transmission between partners. Integrating CVCT services within existing programs can help normalize couples’ testing, facilitate the reduction of stigma, overcome fear of partner reactions, and increase favorable attitudes toward CVCT.

Logistical barriers, reported by one quarter of respondents in Kigali and Lusaka, have been previously reported for individual VCT [11], [39] and CVCT [6] in Africa, although at the time of the survey HIV testing at government facilities was at no-cost. Respondents may have included transport costs and lost wages in their assessment of testing costs [39], [40]. These logistical obstacles highlight the need to increase availability of CVCT services within existing programs, including antenatal, PMTCT, and antiretroviral treatment (ART) platforms, and to bring services closer to clients through mobile, home-based, or community testing initiatives [41], [42], [43], [44], [45], [46]. The integration of couples’ testing in existing HIV programs presents an opportunity to combine treatment, care, and prevention efforts, and to leverage perinatal and heterosexual prevention.

Stigma and partner’s reaction were the most commonly cited barriers to CVCT in this study, and must be addressed in programming and service delivery [21], [40], [47]. Respondents reported receiving information about couples’ testing from a variety of sources, primarily through radio and local health clinics in Kigali and through friends, radio, and television in Lusaka. Mobilization through mass media and facility-based venues may reduce stigma and encourage joint testing. Community-based efforts utilizing health workers, community leaders, faith-based organizations, peer community workers [48], or influential network agents (INA) models [49] should alleviate barriers to seeking CVCT, educate couples on where to go for CVCT services, and emphasize the prevention impact of CVCT.

Differences were observed between respondents in the intervention and control neighborhoods of Kigali. Given that control respondents were more likely to cohabitate, to hear about CVCT from health clinics, to cite treatment and knowledge of one's serostatus as reasons for seeking CVCT, and less likely to fear a partner's reaction, it is reasonable to expect that baseline uptake of CVCT in control neighborhoods would be higher than intervention neighborhoods. Differences observed between intervention and control neighborhoods in Lusaka followed a similar pattern. These differences in baseline characteristics of intervention and control neighborhoods in Kigali and Lusaka may decrease the possible observable effect size of the intervention, but would not falsely overstate the intervention impact.

We acknowledge several limitations of the study which limit generalizability. Because selection of individuals within households was not random, respondents do not represent the general populations of Kigali and Lusaka. However, the sampling of households was random to minimize biases in the selection of households. In making comparisons we must note significant differences between the two cities: Kigali has a smaller population than Lusaka (800,000 *vs*. 1.7 million), and only one local language, Kinyarwanda, with three radio stations broadcasting nationwide, and newspapers and television in Kinyarwanda, English, and French [50], [51], [52]. In contrast, Lusaka has over 70 local dialects from five major language groups, with radio, television and newspapers in English and several local dialects. We have been promoting CVCT in Kigali since 1988 and in Lusaka since 1994. While we did not include the ‘contaminated’ neighborhoods where we had previously worked in our sampling, spillover and/or residual impact from radio and newspaper advertisements is possible, which is expected to have more impact in a smaller city with broader mass media coverage and a longer exposure to the messages. We feel that these findings are relevant to current promotional effects. Given that promotional activities carried out since this survey have lacked large-scale penetration, especially in Lusaka, and since CVCT is not currently a social norm in Zambia, we do not anticipate that awareness of serodiscordance or knowledge and perceptions of CVCT have changed significantly to date. Finally, we did not measure whether study participants had previously tested for HIV, which may be related to knowledge and attitudes towards testing, since collection of this information might be interpreted by participants as asking about their HIV status and be viewed as intrusive. This assumption was deemed to be reasonable given the level of fear and stigma associated with HIV testing.

Individual VCT facilitates access to care, treatment, and support group services, but fails to address mutual disclosure, information on serodiscordance, and counseling support to minimize negative consequences within the couple. Gaps in knowledge about HIV discordance coupled with low disclosure of HIV serostatus to sexual partners remain key impediments to HIV prevention efforts in sub-Saharan Africa. More resources should be directed toward active promotion of CVCT and integration of CVCT into existing antenatal, PMTCT, and ART service platforms to prevent HIV infections in the largest population at risk in sub-Saharan Africa, cohabiting couples. Counseling messages on HIV discordance should be standardized and integrated into VCT programs. Population based health surveys and HIV intervention programs should include couple testing indicators. Given the likely differences from one setting to another, assessments of knowledge of and access to services for CVCT, as well as knowledge of HIV discordance, can help set the stage for the expansion of programming and monitoring and evaluation of couples' testing services.

## Supporting Information

Table S1 Demographic Profile, Knowledge and Perceptions of Couples' VCT^a^ in Intervention and Control Neighborhoods.
(DOC)

Table S2 Knowledge and Perceptions of Couples' VCT^a^ by Cohabitation, Education, and Gender among Kigali Respondents.
(DOC)

Table S3 Knowledge and Perceptions of Couples' VCT^a^ by Cohabitation, Education, and Gender among Lusaka Respondents.
(DOC)
