# Supplementary material for: Knowledge and Perceptions of Couples' Voluntary Counseling and Testing in Urban Rwanda and Zambia: A Cross-Sectional Household Survey
Source: PLoS One. 2011 May 9;6(5):e19573. doi: 10.1371/journal.pone.0019573 (PMC3090401; doi:10.1371/journal.pone.0019573)
Supplement: Table S1 — Demographic Profile, Knowledge and Perceptions of Couples' VCTa in Intervention and Control Neighborhoods. (DOC) [file pone.0019573.s001.doc]

| **TABLE S1. Demographic Profile, Knowledge and Perceptions of Couples' VCT^a^ in Intervention and Control Neighborhoods** | | | | | | | | | | | | | | | |
| --- | --- | --- | --- | --- | --- | --- | --- | --- | --- | --- | --- | --- | --- | --- | --- |
|  | **Kigali** | | **Lusaka** | |  | **Kigali** | | | |  | **Lusaka** | | | |  |
|  | **Total** | | **Total** | |  | **Intervention** | | **Control** | |  | **Intervention** | | **Control** | |  |
|  | **(N = 600)** | | **(N = 603)** | |  | **(N = 400)** | | **(N = 200)** | |  | **(N = 402)** | | **(N = 201)** | |  |
|  | **N** | **%** | **N** | **%** | **p-value** | **N** | **%** | **N** | **%** | **p-value** | **N** | **%** | **N** | **%** | **p-value** |
| **Demographic characteristics** |  |  |  |  |  |  |  |  |  |  |  |  |  |  |  |
| Age (mean and standard deviation) | 29 | 9 | 29 | 10 |  | 29 | 9 | 30 | 9 |  | 28 | 11 | 31 | 9 | *** |
| Education |  |  |  |  | *** |  |  |  |  |  |  |  |  |  | *** |
| Less than Secondary | 415 | 69% | 242 | 40% |  | 277 | 69% | 138 | 69% |  | 181 | 45% | 61 | 30% |  |
| Secondary or higher | 185 | 31% | 337 | 56% |  | 123 | 31% | 62 | 31% |  | 204 | 51% | 133 | 66% |  |
| Living Situation |  |  |  |  |  |  |  |  |  | * |  |  |  |  | *** |
| Cohabiting | 368 | 61% | 341 | 57% |  | 234 | 59% | 134 | 67% |  | 207 | 51% | 134 | 67% |  |
| Non-cohabiting | 232 | 39% | 259 | 43% |  | 166 | 42% | 66 | 33% |  | 194 | 48% | 65 | 32% |  |
| **Knowledge and Perceptions** |  |  |  |  |  |  |  |  |  |  |  |  |  |  |  |
| Know of a place to test for HIV | 502 | 84% | 416 | 69% | *** | 334 | 84% | 168 | 84% |  | 257 | 64% | 159 | 79% | *** |
| Where to go to test for HIV |  |  |  |  |  |  |  |  |  |  |  |  |  |  |  |
| Hospital or Health Center | 387 | 65% | 217 | 36% | *** | 268 | 67% | 119 | 60% |  | 165 | 41% | 52 | 26% | *** |
| VCT Center | 103 | 17% | 197 | 33% | *** | 62 | 16% | 41 | 21% |  | 91 | 23% | 106 | 53% | *** |
| Blood Bank/Family Planning Center/Other | 12 | 2% | 2 | 0.3% | * | 4 | 1% | 8 | 4% | * | 1 | 0% | 1 | 0% |  |
| Know the name of a nearby place to test for HIV | 472 | 79% | 336 | 56% | *** | 320 | 80% | 152 | 76% |  | 184 | 46% | 152 | 76% | *** |
| Heard/know about VCT for couples | 561 | 94% | 406 | 67% | *** | 377 | 94% | 184 | 92% |  | 267 | 66% | 139 | 69% |  |
| How, where, or from who they heard about CVCT | | | | | | | | | | | | | | | |
| Radio | 384 | 64% | 186 | 31% | *** | 261 | 65% | 123 | 62% |  | 110 | 27% | 76 | 38% | ** |
| Television | 48 | 8% | 154 | 26% | *** | 26 | 7% | 22 | 11% |  | 85 | 21% | 69 | 34% | *** |
| Newspaper | 45 | 8% | 41 | 7% |  | 28 | 7% | 17 | 9% |  | 22 | 5% | 19 | 9% |  |
| Local health clinic | 130 | 22% | 84 | 14% | *** | 100 | 25% | 30 | 15% | ** | 61 | 15% | 23 | 11% |  |
| Friend | 76 | 13% | 209 | 35% | *** | 48 | 12% | 28 | 14% |  | 138 | 34% | 71 | 35% |  |
| Neighbor | 66 | 11% | 76 | 13% |  | 38 | 10% | 28 | 14% |  | 49 | 12% | 27 | 13% |  |
| Family | 77 | 13% | 44 | 7% | ** | 53 | 13% | 24 | 12% |  | 21 | 5% | 23 | 11% | ** |
| Church | 80 | 13% | 56 | 9% | * | 43 | 11% | 37 | 19% | ** | 32 | 8% | 24 | 12% |  |
| It is possible for a married/cohabiting couple to be HIV discordant | 498 | 83% | 261 | 43% | *** | 335 | 84% | 163 | 82% |  | 162 | 40% | 99 | 49% | * |
| A person testing alone should share HIV results with partner | 541 | 90% | 464 | 77% | *** | 360 | 90% | 181 | 91% |  | 318 | 79% | 146 | 73% |  |
| Opinion about married/cohabiting couples testing together for HIV | | | | | | | | | | | | | | | |
| Couples joint HIV testing is good | 573 | 96% | 432 | 72% | *** | 382 | 96% | 191 | 96% |  | 283 | 70% | 149 | 74% |  |
| Couples joint HIV testing is not good | 27 | 5% | 109 | 18% | *** | 18 | 5% | 9 | 5% |  | 71 | 18% | 38 | 19% |  |
| No opinion | 0 | 0% | 61 | 10% | *** | 0 | 0% | 0 | 0% |  | 48 | 12% | 13 | 6% | * |
| Willingness to test with spouse |  |  |  |  |  |  |  |  |  |  |  |  |  |  |  |
| My partner and I can test together | 548 | 91% | 282 | 47% | *** | 367 | 92% | 181 | 91% |  | 183 | 46% | 99 | 49% |  |
| I can test alone but not with my partner | 21 | 4% | 38 | 6% | * | 14 | 4% | 7 | 4% |  | 26 | 6% | 12 | 6% |  |
| I am not interested in testing for HIV | 4 | 1% | 52 | 9% | *** | 1 | 0% | 3 | 2% |  | 29 | 7% | 23 | 11% |  |
| I prefer not to discuss HIV testing | 0 | 0% | 13 | 2% | ** | 0 | 0% | 0 | 0% |  | 8 | 2% | 5 | 2% |  |
| Couples testing together is not good because | | | | | | | | | | | | | | | |
| It may break up the family | 22 | 4% | 57 | 9% | *** | 16 | 4% | 6 | 3% |  | 36 | 9% | 21 | 10% |  |
| It may lead to depression | 4 | 1% | 39 | 6% | *** | 1 | 0% | 3 | 2% |  | 25 | 6% | 14 | 7% |  |
| It is not important, it is God's will | 1 | 0% | 11 | 2% | ** | 1 | 0% | 0 | 0% |  | 9 | 2% | 2 | 1% |  |
| **Facilitators and Barriers to seeking CVCT services** | | | | | | | | | | | | | | | |
| What is the major reason preventing couples from getting tested for HIV together? | | | | | | | | | | | | | | | |
| Stigma | 172 | 29% | 306 | 51% | *** | 112 | 28% | 60 | 30% |  | 181 | 45% | 125 | 62% | *** |
| Partner reaction | 243 | 41% | 147 | 24% | *** | 176 | 44% | 67 | 34% | * | 94 | 23% | 53 | 26% |  |
| Distance to health facility or cost of test | 139 | 23% | 62 | 10% | *** | 87 | 22% | 52 | 26% |  | 56 | 14% | 6 | 3% | *** |
| Duration of the test or taking of blood | 37 | 6% | 51 | 8% |  | 23 | 6% | 14 | 7% |  | 40 | 10% | 11 | 5% | * |
| Reasons couples may seek CVCT services: | | | | | | | | | | | | | | | |
| Treatment possibilities | 182 | 30% | 156 | 26% |  | 109 | 27% | 73 | 37% | * | 108 | 27% | 48 | 24% |  |
| To prevent vertical transmission | 152 | 25% | 86 | 14% | *** | 103 | 26% | 49 | 25% |  | 53 | 13% | 33 | 16% |  |
| To prevent transmission between partners | 152 | 25% | 87 | 14% | *** | 107 | 27% | 45 | 23% |  | 48 | 12% | 39 | 19% | * |
| To know one's HIV test results | 282 | 47% | 548 | 91% | *** | 162 | 41% | 120 | 60% | *** | 368 | 92% | 180 | 90% |  |
| To plan for family's future | 199 | 33% | 214 | 35% |  | 151 | 38% | 48 | 24% | *** | 140 | 35% | 74 | 37% |  |
| **p*<0.05; ***p*<0.01; ****p*<0.001  ^a^(C)VCT: (couples’) voluntary counseling and testing; Note: Totals do not always add to 100% due to missing values. | | | | | | | | | | | | | | | |
