# Supplementary material for: Knowledge and Perceptions of Couples' Voluntary Counseling and Testing in Urban Rwanda and Zambia: A Cross-Sectional Household Survey
Source: PLoS One. 2011 May 9;6(5):e19573. doi: 10.1371/journal.pone.0019573 (PMC3090401; doi:10.1371/journal.pone.0019573)
Supplement: Table S2 — Knowledge and Perceptions of Couples' VCTa by Cohabitation, Education, and Gender among Kigali Respondents. (DOC) [file pone.0019573.s002.doc]

| **TABLE S2. Knowledge and Perceptions of Couples' VCT^a^ by Cohabitation, Education, and Gender among Kigali Respondents** | | | | | | | | | | | | | | | |
| --- | --- | --- | --- | --- | --- | --- | --- | --- | --- | --- | --- | --- | --- | --- | --- |
|  | **Cohabitation** | | | |  |  | **Education** | | | | **Gender** | | | |  |
|  | **Cohabiting** | | **Non-cohabiting** | |  | | **Less than Secondary** | | **Secondary or higher** | | **Men** | | **Women** | |  |
|  | **(N=368)** | | **(N= 232)** | |  | | **(N = 415)** | | **(N = 185)** | | **(N = 300)** | | **(N = 300)** | |  |
|  | **N** | **%** | **N** | **%** | **p value** | **N** | **%** | **N** | **%** | **p value** | **N** | **%** | **N** | **%** | **p value** |
| **Knowledge and Perceptions** |  |  |  |  |  |  |  |  |  |  |  |  |  |  |  |
| Know of a place to test for HIV | 327 | 89% | 175 | 75% | *** | 328 | 79% | 174 | 94% | *** | 248 | 83% | 253 | 84% |  |
| Where to go to test for HIV |  |  |  |  |  |  |  |  |  |  |  |  |  |  |  |
| Hospital or Health Center | 249 | 68% | 138 | 59% | * | 260 | 63% | 127 | 69% |  | 177 | 59% | 210 | 70% | ** |
| VCT Center | 69 | 19% | 34 | 15% |  | 64 | 15% | 39 | 21% |  | 62 | 21% | 41 | 14% | * |
| Blood Bank/Family Planning Center/Other | 9 | 2% | 3 | 1% |  | 4 | 1% | 8 | 4% | * | 9 | 3% | 3 | 1% |  |
| Know the name(s) of a place near you to test | 309 | 84% | 163 | 70% | *** | 304 | 73% | 168 | 91% | *** | 228 | 76% | 244 | 81% |  |
| Heard/know about VCT for couples | 348 | 95% | 213 | 92% |  | 383 | 92% | 178 | 96% |  | 282 | 94% | 279 | 93% |  |
| How, where, or from who they heard about CVCT | | | | | | | | | | | | | | | |
| Radio | 242 | 66% | 142 | 61% |  | 253 | 61% | 131 | 71% | * | 205 | 68% | 179 | 60% | * |
| Television | 31 | 8% | 17 | 7% |  | 16 | 4% | 32 | 17% | *** | 26 | 9% | 22 | 7% |  |
| Newspaper | 31 | 8% | 14 | 6% |  | 14 | 3% | 31 | 17% | *** | 27 | 9% | 18 | 6% |  |
| Local health clinic | 101 | 27% | 29 | 13% | *** | 81 | 20% | 49 | 26% |  | 54 | 18% | 76 | 25% | * |
| Friend | 38 | 10% | 38 | 16% | * | 63 | 15% | 23 | 12% |  | 34 | 11% | 42 | 14% |  |
| Neighbor | 38 | 10% | 28 | 12% |  | 49 | 12% | 17 | 9% |  | 31 | 10% | 35 | 12% |  |
| Family | 38 | 10% | 39 | 17% | * | 50 | 12% | 27 | 15% |  | 36 | 12% | 41 | 14% |  |
| Church | 50 | 14% | 30 | 13% |  | 59 | 14% | 21 | 11% |  | 35 | 12% | 45 | 15% |  |
| It is possible for a married/cohabiting couple to be HIV discordant | 310 | 84% | 188 | 81% |  | 332 | 80% | 166 | 90% | ** | 245 | 82% | 253 | 84% |  |
| A person testing alone should share HIV results with partner | 331 | 90% | 210 | 91% |  | 376 | 91% | 165 | 89% |  | 276 | 92% | 265 | 88% |  |
| Opinion about married/cohabiting couples testing together for HIV |  |  |  |  | * |  |  |  |  | * |  |  |  |  |  |
| Couples joint HIV testing is good | 357 | 97% | 216 | 93% |  | 391 | 94% | 182 | 98% |  | 287 | 96% | 286 | 95% |  |
| Couples joint HIV testing is not good | 11 | 3% | 16 | 7% |  | 24 | 6% | 3 | 2% |  | 13 | 4% | 14 | 5% |  |
| No opinion | 0 | 0% | 0 | 0% |  | 0 | 0% | 0 | 0% |  | 0 | 0% | 0 | 0% |  |
| Willingness to test with spouse |  |  |  |  |  |  |  |  |  |  |  |  |  |  |  |
| My partner and I can test together | 342 | 93% | 206 | 89% |  | 377 | 91% | 171 | 92% |  | 273 | 91% | 275 | 92% |  |
| I can test alone but not with my partner | 12 | 3% | 9 | 4% |  | 10 | 2% | 11 | 6% | * | 12 | 4% | 9 | 3% |  |
| I am not interested in testing for HIV | 3 | 1% | 1 | 0% |  | 4 | 1% | 0 | 0% |  | 2 | 1% | 2 | 1% |  |
| I prefer not to discuss HIV testing | 0 | 0% | 0 | 0% |  | 0 | 0% | 0 | 0% |  | 0 | 0% | 0 | 0% |  |
| Couples testing together is not good because: |  |  |  |  |  |  |  |  |  |  |  |  |  |  |  |
| It may break up the family | 9 | 2% | 13 | 6% | * | 19 | 5% | 3 | 2% |  | 10 | 3% | 12 | 4% |  |
| It may lead to depression | 1 | 0% | 3 | 1% |  | 4 | 1% | 0 | 0 |  | 3 | 1% | 1 | 0% |  |
| It is not important, it is God's will | 1 | 0% | 0 | 0% |  | 1 | 0% | 0 | 0 |  | 0 | 0% | 1 | 0% |  |
| **Facilitators and Barriers to seeking CVCT services** | | | | | | | | | | | | | | | |
| What is the major reason preventing couples from getting tested for HIV together? | | | | | | | | | | | | | | |  |
| Stigma | 90 | 24% | 82 | 35% | ** | 111 | 27% | 61 | 33% |  | 101 | 34% | 71 | 24% | ** |
| Partner reaction | 162 | 44% | 81 | 35% | * | 157 | 38% | 86 | 46% | * | 106 | 35% | 137 | 46% | ** |
| Distance to health facility/cost of test | 86 | 23% | 53 | 23% |  | 115 | 28% | 24 | 13% | *** | 74 | 25% | 65 | 22% |  |
| Duration of the test/taking of blood | 24 | 7% | 13 | 6% |  | 24 | 6% | 13 | 7% |  | 14 | 5% | 23 | 8% |  |
| Reasons couples may seek CVCT services: | | | | | | | | | | | | | | |  |
| Treatment possibilities | 116 | 32% | 66 | 28% |  | 126 | 30% | 56 | 30% |  | 86 | 29% | 96 | 32% |  |
| To prevent vertical transmission | 98 | 27% | 54 | 23% |  | 97 | 23% | 55 | 30% |  | 78 | 26% | 74 | 25% |  |
| To prevent HIV transmission between partners | 87 | 24% | 65 | 28% |  | 105 | 25% | 47 | 25% |  | 79 | 26% | 73 | 24% |  |
| To know one's HIV test results | 166 | 45% | 116 | 50% |  | 199 | 48% | 83 | 45% |  | 137 | 46% | 145 | 48% |  |
| To plan for family's future | 130 | 35% | 69 | 30% |  | 131 | 32% | 68 | 37% |  | 105 | 35% | 94 | 31% |  |
| *p<0.05; **p<0.01; ***p<0.001  ^a^(C)VCT: (couples’) voluntary counseling and testing; Note: Totals do not always add up due to missing values. | | | | | | | | | | | | | | | |
