# Supplementary material for: Knowledge and Perceptions of Couples' Voluntary Counseling and Testing in Urban Rwanda and Zambia: A Cross-Sectional Household Survey
Source: PLoS One. 2011 May 9;6(5):e19573. doi: 10.1371/journal.pone.0019573 (PMC3090401; doi:10.1371/journal.pone.0019573)
Supplement: Table S3 — Knowledge and Perceptions of Couples' VCTa by Cohabitation, Education, and Gender among Lusaka Respondents. (DOC) [file pone.0019573.s003.doc]

| **TABLE S3. Knowledge and Perceptions of Couples' VCT^a^ by Cohabitation, Education, and Gender among Lusaka Respondents** | | | | | | | | | | | | | | | |
| --- | --- | --- | --- | --- | --- | --- | --- | --- | --- | --- | --- | --- | --- | --- | --- |
|  | **Cohabitation** | | | |  | **Education** | | | |  | **Gender** | | | |  |
|  | **Cohabiting** | | **Non-cohabiting** | |  | **Less than Secondary** | | **Secondary or higher** | |  | **Men** | | **Women** | |  |
|  | **(N = 341)** | | **(N = 259)** | |  | **(N = 242)** | | **(N = 337)** | |  | **(N = 300)** | | **(N = 303)** | |  |
|  | **N** | **%** | **N** | **%** | **p value** | **N** | **%** | **N** | **%** | **p value** | **N** | **%** | **N** | **%** | **p value** |
| **Knowledge and Perceptions** |  |  |  |  |  |  |  |  |  |  |  |  |  |  |  |
| Know of a place to test for HIV | 243 | 71% | 172 | 66% |  | 132 | 55% | 266 | 79% | *** | 199 | 66% | 217 | 72% |  |
| Where to go to test for HIV |  |  |  |  |  |  |  |  |  |  |  |  |  |  |  |
| Hospital or Health Center | 129 | 38% | 87 | 34% |  | 78 | 32% | 130 | 39% |  | 96 | 32% | 121 | 40% | * |
| VCT Center | 84 | 25% | 113 | 44% | *** | 54 | 22% | 134 | 40% | *** | 102 | 34% | 95 | 31% |  |
| Blood Bank/Family Planning Center/Other | 1 | 0% | 1 | 0% |  | 0 | 0% | 2 | 1% |  | 1 | 0% | 1 | 0% |  |
| Know the name(s) of a place near you to test | 202 | 59% | 133 | 51% |  | 102 | 42% | 222 | 66% | *** | 164 | 55% | 172 | 57% |  |
| Know about VCT for couples | 231 | 68% | 175 | 68% |  | 151 | 62% | 238 | 71% | * | 201 | 67% | 205 | 68% |  |
| How, where, or from who they heard about CVCT | | | | | | | | | | | | | | | |
| Radio | 113 | 33% | 73 | 28% |  | 65 | 27% | 115 | 34% |  | 101 | 34% | 85 | 28% |  |
| Television | 92 | 27% | 62 | 24% |  | 48 | 20% | 104 | 31% | ** | 76 | 25% | 78 | 26% |  |
| Newspaper | 28 | 8% | 13 | 5% |  | 11 | 5% | 29 | 9% |  | 27 | 9% | 14 | 5% | * |
| Local health clinic | 57 | 17% | 27 | 10% | * | 34 | 14% | 48 | 14% |  | 29 | 10% | 55 | 18% | ** |
| Friend | 120 | 35% | 89 | 34% |  | 77 | 32% | 121 | 36% |  | 110 | 37% | 99 | 33% |  |
| Neighbor | 39 | 11% | 37 | 14% |  | 39 | 16% | 36 | 11% |  | 35 | 12% | 41 | 14% |  |
| Family | 24 | 7% | 20 | 8% |  | 15 | 6% | 27 | 8% |  | 22 | 7% | 22 | 7% |  |
| Church | 33 | 10% | 23 | 9% |  | 19 | 8% | 37 | 11% |  | 23 | 8% | 33 | 11% |  |
| It is possible for a married/cohabiting couple to be HIV discordant | 153 | 45% | 106 | 41% |  | 83 | 34% | 164 | 49% | *** | 113 | 38% | 148 | 49% | ** |
| A person testing alone should share HIV results with partner | 267 | 78% | 195 | 75% |  | 180 | 74% | 268 | 80% |  | 219 | 73% | 245 | 81% | * |
| Opinion about married/cohabiting couples testing together for HIV | | | | | | | | | | | | | | | |
| Couples joint HIV testing is good | 254 | 74% | 176 | 68% |  | 158 | 65% | 258 | 77% | ** | 214 | 71% | 218 | 72% |  |
| Couples joint HIV testing is not good | 67 | 20% | 42 | 16% |  | 54 | 22% | 49 | 15% | * | 57 | 19% | 52 | 17% |  |
| No opinion | 19 | 6% | 41 | 16% | *** | 30 | 12% | 29 | 9% |  | 27 | 9% | 34 | 11% |  |
| Willingness to test with spouse |  |  |  |  |  |  |  |  |  |  |  |  |  |  |  |
| My partner and I can test together | 192 | 56% | 90 | 35% | *** | 95 | 39% | 177 | 53% | ** | 134 | 45% | 148 | 49% |  |
| I can test alone but not with my partner | 23 | 7% | 15 | 6% |  | 17 | 7% | 20 | 6% |  | 15 | 5% | 23 | 8% |  |
| I am not interested in testing for HIV | 31 | 9% | 21 | 8% |  | 28 | 12% | 24 | 7% |  | 26 | 9% | 26 | 9% |  |
| I prefer not to discuss HIV testing | 7 | 2% | 6 | 2% |  | 8 | 3% | 5 | 1% |  | 3 | 1% | 10 | 3% |  |
| Couples testing together is not good because: | | | | | | | | | | | | | | |  |
| It may break up the family | 36 | 11% | 21 | 8% |  | 28 | 12% | 26 | 8% |  | 31 | 10% | 26 | 9% |  |
| It may lead to depression | 22 | 6% | 17 | 7% |  | 16 | 7% | 21 | 6% |  | 19 | 6% | 20 | 7% |  |
| It is not important, it is God's will | 8 | 2% | 3 | 1% |  | 9 | 4% | 3 | 1% | * | 6 | 2% | 5 | 2% |  |
| **Facilitators and Barriers to seeking CVCT services** | | | | | | | | | | | | | | | |
| What is the major reason preventing couples from getting tested for HIV together? | | | | | | | | | | | | | | |  |
| Stigma | 166 | 49% | 138 | 53% |  | 117 | 48% | 181 | 54% |  | 139 | 46% | 167 | 55% | * |
| Partner reaction | 85 | 25% | 61 | 24% |  | 58 | 24% | 82 | 24% |  | 80 | 27% | 67 | 22% |  |
| Distance to health facility or cost of test | 34 | 10% | 28 | 11% |  | 24 | 10% | 33 | 10% |  | 42 | 14% | 20 | 7% | ** |
| Duration of the test or taking of blood | 32 | 9% | 19 | 7% |  | 24 | 10% | 24 | 7% |  | 20 | 7% | 31 | 10% |  |
| Reasons couples may seek CVCT services |  |  |  |  |  |  |  |  |  |  |  |  |  |  |  |
| Treatment possibilities | 91 | 27% | 65 | 25% |  | 71 | 29% | 79 | 23% |  | 73 | 24% | 83 | 27% |  |
| To prevent vertical transmission | 56 | 16% | 30 | 12% |  | 30 | 12% | 51 | 15% |  | 41 | 14% | 45 | 15% |  |
| To prevent HIV transmission between partners | 54 | 16% | 33 | 13% |  | 31 | 13% | 50 | 15% |  | 53 | 18% | 34 | 11% | * |
| To know one's HIV test results | 312 | 91% | 235 | 91% |  | 219 | 90% | 306 | 91% |  | 271 | 90% | 277 | 91% |  |
| To plan for family's future | 139 | 41% | 73 | 28% | ** | 85 | 35% | 123 | 36% |  | 102 | 34% | 112 | 37% |  |
